# Stability of volcanic ash aggregates and break-up processes

**DOI:** 10.1038/s41598-017-07927-w

**Published:** 2017-08-07

**Authors:** Sebastian B. Mueller, Ulrich Kueppers, Jonathan Ametsbichler, Corrado Cimarelli, Jonathan P. Merrison, Matthieu Poret, Fabian B. Wadsworth, Donald B. Dingwell

**Affiliations:** 1 Ludwig-Maximilians-Universität München, Munich, Germany; 2 Aarhus Universitet, Aarhus, Denmark; 3 Istituto Nazionale di Geofisica e Vulcanologia, Bologna section, Bologna, Italy

## Abstract

Numerical modeling of ash plume dispersal is an important tool for forecasting and mitigating potential hazards from volcanic ash erupted during explosive volcanism. Recent tephra dispersal models have been expanded to account for dynamic ash aggregation processes. However, there are very few studies on rates of disaggregation during transport. It follows that current models regard ash aggregation as irrevocable and may therefore overestimate aggregation-enhanced sedimentation. In this experimental study, we use industrial granulation techniques to artificially produce aggregates. We subject these to impact tests and evaluate their resistance to break-up processes. We find a dependence of aggregate stability on primary particle size distribution and solid particle binder concentration. We posit that our findings could be combined with eruption source parameters and implemented in future tephra dispersal models.

## Introduction

Numerous investigations, using field^1–5^, numerical^6, 7^ and experimental^8–12^ approaches have extended our understanding of the generation of volcanic ash aggregates. The control of volcanic ash aggregation on ash plume dispersal has also been demonstrated by field studies^13–15^ and is now a common component in numerical modeling of volcanic ash dispersal^16–19^. However, an understanding of aggregate preservation potential during transport and sedimentation processes is not yet fully understood. Ash is exposed to strongly variable transport conditions that may control aggregation rates (e.g. wind speed, temperature, humidity, acidity, glass content of the ash, particle-particle interaction rates). Nevertheless, the same factors can also control aggregate preservation potential. Disaggregation processes resulting from the elastic mechanical stresses associated with particle-particle interactions may occur both during transport (aggregate-aggregate or aggregate-particle) as well as during sedimentation (aggregate-substrate). There are two key controls on aggregate stability: (1) the properties of the ash particles that form aggregates (i.e. primary particle size, morphology and the aggregate binder agent^20–22^) and (2) aggregate size, shape and roughness. Aggregates fail to remain intact and cohesive if extrinsic elastic stress is higher than the tensile strength of inter-particle contact areas. Analysis of large volcanic aggregates (i.e. mm- to cm-size) from several locations has shown secondary mineral phases like NaCl, MgSO_4_ or CaSO_4_^2, 8, 12, 23–25^ that act as binding agents between particles_._ Crack initiation in such solid salt bridges may lead to either internal failure of the solid bridge (cohesive failure) or failure of the contact line between solid bridge and particle (adhesive failure^26^). Depending on the initial impact energy, particles may be chipped off from the aggregate surface (low impact energy), the aggregate may fragment into several parts (moderate impact energy) or the aggregate may wholly disaggregate into primary particles (high impact energy^27^). Any size reduction process will liberate individual ash particles that subsequently respond in their dispersal behavior based on the aerodynamic properties of single ash shards whose density and drag coefficients differ from those of the prior aggregates. Clearly then, ash aggregation cannot be solely considered as irreversible process that progressively contributes to depletion of airborne ash. The probability of disaggregation processes and resultant influence of locally increased ash concentrations on bulk ash plume dispersal remains unexplored and is not explicitly implemented in tephra dispersal models.

Recent experimental studies have shown the influence of density instabilities and ash particle concentration (loose, not aggregated) on dispersal and settling behavior (distance from vent and velocity) of volcanic ash^28, 29^. From a computational point of view, model results of tephra dispersal and deposition is crucially modified by ash aggregation processes. Neglecting aggregation within a tephra dispersal model may lead to a tephra loading underestimate in proximal area (tens to hundreds of kilometers of distance from the vent) and an overestimate in distal regions^3, 30^. The removal of ash particles from the plume in aggregation events, and the re-release of those ash particles during disaggregation events affects the dispersal and the tephra fallout deposit thickness variations down-wind. Although a variable ash dispersal pattern has been observed and reported for several eruptions and attributed to aggregation processes^7, 31–33^, this is not necessarily reflected in the fall deposits of the eruption, because disaggregation during deposition can remove the evidence that particles aggregated in the first place^34^. While the latest models of ash dispersal take ash aggregation into account by solving for advection-diffusion-sedimentation equations in defined meteorological conditions and using input eruption source parameters (e.g., eruption duration, column height, total erupted mass, mass eruption rate, and total grainsize distribution)^19, 33^, no plume model incorporates post-aggregation disaggregation processes. Such plume models incorporate aggregation as an effective aggregated class of particles in the plume (characterized by a diameter *d*_*a*_ and a density *ρ*_*a*_) in different ways. For example, the Cornell model^35^ fills the class with 50% of the 63–44 µm ash particles, 75% of the 44–31 µm and 90% of the smaller than 31 µm. The Sulpizio model^36^ considers a constant aggregated fraction defined by the user. And the Costa *et al*. model^17, 18^ is more complex and solves for the first order of the Smoluchowski equation^37^ to estimate the fractions of each gain-size class to remove from the primary particle classes. Software packages such as FPlume^19^ and FALL3D^16, 38, 39^ have implemented each of these techniques. From these constraints, among the eruption source parameters, aggregation processes are likely to affect the total grain size distribution most substantially, and especially the fine-ash tail of the distribution. This highlights the need to assess the total size distribution, which is representative of bulk tephra deposits.

It’s clear that aggregation models are incorporated into plume models. But it is also important to implement disaggregation processes based on both observational and experimental data. Aggregate growth and stability characteristics can be estimated by coupling observations from deposits with *in-situ* observations during eruptions through the application of monitored eruption source parameters^40^, such as total grain size distribution or mass eruption rate. We can envisage end member scenarios for the aggregation-disaggregation process in plumes. First, efficient aggregation occurs. This can be because aggregation is efficient while disaggregation is inefficient such that particle fall deposits are modified by the progressive coarsening of the plume load. Second, no net aggregation occurs, which can be because aggregation is inefficient or because disaggregation is more efficient than aggregation. This scenario would result in particle size classes that are either unaffected during classic plume transport, or they are variably aggregated and disaggregated in-plume causing additional complexity in ash dispersal patterns.

Here, we present the results of an experimental campaign in which aggregates, bound by salt bridges at particle-particle contact points^41^, have been subjected to impact events at a range of constrained energies. We identify the failure modes and estimate the strength of the aggregates of particles which are crucial parameters for future incorporation of disaggregation processes into tephra dispersal models.

## Methods

### Production of sample materials

Experimentally-generated aggregates of 1) soda-lime silicate glass beads and 2) phonolitic Laacher See volcanic ash (Eifel, Germany), were used for the experiments; the granulometry of selected samples was determined using a Coulter LS-230 laser diffraction particle size analyser (Fraunhofer optical model, imaginary/real refractive indices of 0.001/1.52 for glass beads and 0.1/1.52 for volcanic ash; see Table 1 and Supplementary Material for detailed grain size distribution of experimental materials). Aggregates were produced at *Glatt Ingenieurtechnik GmbH*, Weimar, Germany, by applying fluidization bed techniques with the *Glatt ProCell Lab* ^®^. Particle aggregation was achieved in two steps. First, particles were placed in a vessel and transformed from a deposited state at rest to a fluid-like state in motion through an upwards directed gas-stream, generating a fluidized bed. This lead to effective particle concentrations of 0.003 g cm^−3^, which relates to dilute and downwind areas of volcanic plumes or lofted plumes coincident with pyroclastic density currents. Humidity was controlled by spraying NaCl-H_2_O mixture of various concentration into the fluidized sample via a nozzle (1.0 bar pressure and 8 ml.min^−1^ spray rate) at low spray rate, the NaCl brine is only wetting the particle surfaces, but did not lead to aggregation. At well-controlled temperatures of 25–110 °C, evaporation of the liquid resulted in precipitation of NaCl crystals on particle surfaces with regular, isotropic distribution. Then, de-ionized H_2_O was sprayed into the fluidized bed at a significantly higher spray-rate of 50 ml.min^−1^. Aerosolized liquid droplets deposited on particle surfaces and partially dissolved the previously deposited NaCl crystals. The high amount of liquid in the second step allowed for particles to cluster for longer periods and for aggregates to grow bigger. Capillary forces allowed for the movement of the surficial NaCl brine to particle-particle contact points^41^. Upon drying, solid NaCl bridges crystallized and cemented the aggregates. Aggregates strong enough to survive the drying and collection process were able to be collected and analyzed for this study. (See ref. 12 for a detailed description of the aggregate production process). Spraying brine liquids simplifies and accelerates the aggregation. When water or acid aerosols condense on ash particles, leaching will cause various elements to be transported to the surface of the grains where they will form precipitates and bind aggregates. While these two processes (acid-driven leaching and salt precipitation and brine evaporation driven salt precipitation) are subtly different, they both produce salt-bound aggregates.

**Table 1:** Overview of physical characteristics of artificially produced aggregates used for impact experiments.

Primary particle material	Primary particle size distribution	min/max/mode [µm]	NaCl [g kg^−1^]
Laacher See Ash	<40 µm	0.41/101.1/24.95	5
Laacher See Ash	<40 µm	0.41/101.1/24.95	10
Laacher See Ash	<40 µm	0.41/101.1/24.95	20
Laacher See Ash	40–90 µm	0.87/146.8/76.42	20
Laacher See Ash	<90 µm	0.45/133.7/76.42	10
Laacher See Ash	<90 µm	0.45/133.7/76.42	15
Laacher See Ash	<90 µm	0.45/133.7/76.42	20
Soda-lime glass beads	<50 µm	0.41/92.09/43.67	5
Soda-lime glass beads	<50 µm	0.41/92.09/43.67	15
Soda-lime glass beads	40–70 µm	22.73/121.81/57.77	2
Soda-lime glass beads	40–70 µm	22.73/121.81/57.77	5
Soda-lime glass beads	40–70 µm	22.73/121.81/57.77	15
Soda-lime glass beads	<70 µm	4.24/101.1/57.77	2
Soda-lime glass beads	<70 µm	4.24/101.1/57.77	5
Soda-lime glass beads	<70 µm	4.24/101.1/57.77	15

Several types of aggregates can be produced by the above method (Table 1). Glass bead aggregates are composed of primary particle sizes of <50 µm, 40–70 µm or <70 µm and are bound with NaCl concentrations of 2, 5 or 15 g.kg^−1^, respectively. Volcanic ash aggregates are comprised of primary particle sizes <40 µm, 40–90 µm or <90 µm and are bound with NaCl concentrations of 5, 10, 15 or 20 g.kg^−1^, respectively. Efficient binding of volcanic ash was found to be impossible at concentrations of 2 g.kg^−1^ due to the higher specific surface area of the starting material^12^. For further experimental details, please refer to ref. 12, 41. Salt concentrations up to a few g.kg^−1^ are likely to be found on ash surfaces that were transported in volcanic plumes of magmatic eruptions. Concentrations of up to 60 g.kg^−1^ have been described for ash particles related to hydrothermal eruptions^42^.

NaCl concentration (g.kg^−1^) of artificial aggregates used for stability experiments were determined by aqueous leaching. The leaching protocol requires a solid solution mass ratio of 1:10 and measured the effective NaCl concentration of particle surfaces via electrical conductivity measurements with an *inoLab Cond 730* ^*®*^, manufactured by *Wissenschaftliche Technische Werkstätten GmbH*, Germany. The instrument was calibrated using H_2_O-NaCl solutions of known concentration. NaCl coating of particle surfaces and hence NaCl concentrations of *ProCell Lab* ^*®*^ aggregates are reproducible within 3% error^12^. Scanning Electron Microscopy (SEM) was carried out at LMU Munich using a *Hitachi SU 5000* and at the HT-HP lab of INGV Rome, using a *JEOL JSM-6500F*.

### Impact testing

**Figure 1:**
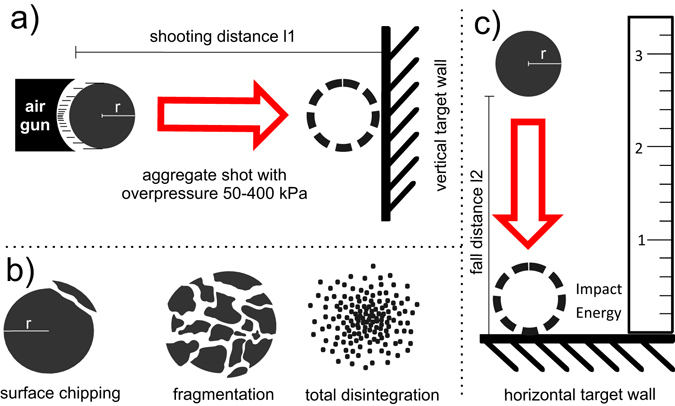
(**a**) Pressurized air-gun setup at Aarhus University, Denmark. Aggregates were shot with overpressure against a vertical metal wall. Applied overpressures ranged between 0.5 and 4 bar, resulting in impact velocities of 2.9–7.8 m.s^−1^. (**b**) Impact setup at LMU Munich, Germany. Aggregates were dropped from heights between 5 and 200 cm onto a metal plate. Impact speed was monitored with a high-speed camera and used to calculate impact energy. (**c**) Modes of break-up that were observed throughout experiments: surface chipping (<10 wt% loss of material from parent aggregate), fragmentation (10–90 wt% material loss from parent aggregate) and total disintegration (>90 wt% material loss from parent aggregate).

Two experimental setups were designed to investigate the modes of breaking behavior of aggregates and the aggregate strength. Setup 1 was built at the *Mars Laboratory Simulation Laboratory* at Aarhus University, Denmark. An over-pressurized nozzle (50–400 kPa) propelled individual volcanic ash aggregates (particle sizes <90 µm with 20 g.kg^−1^ NaCl) against a vertical target wall aligned perpendicular to the aggregate flight path. The impact was recorded with a *Phantom v710* high-speed camera (Fig. 1a). Varying the overpressure condition at the nozzle, we investigated a range of impact velocities for which we observed different modalities of aggregate breakup upon impact such as surface chipping, fragmentation and total disintegration (Fig. 1b). Setup 2, built at LMU Munich, Germany, aimed at investigating the aggregate strength by free-fall experiments. Individual aggregates were dropped from a height *h* onto a metal plate (Fig. 1c). These experiments were designed to evaluate the effect of particle size distribution (PSD), shape and surface morphology of primary particles and binder concentration on aggregate stability. We performed these experiments on artificial aggregates only as young natural ash aggregates are rare and even the youngest^43^ have likely already undergone further post-depositional (re-)crystallization, e.g. by growth of zeolites.

Following a pre-established framework^44^ allowed us to parameterize the potential, kinetic and loss-during-flight (atmospheric) energies of particles during both horizontal (Setup 1) and vertical (Setup 2) experiments. For a quantitative approach, potential energies *E*_p_ of the aggregates were calculated and used to estimate kinetic energies *E*_K_ of the samples during impact. During fall, samples will lose energy due to drag, an energy loss which we term a loss energy *E*_L_. These energy scalings are given by Eq. 1:

1 $$\begin{array}{c}{E}_{{\rm{P}}}={m}_{i}gh\\ {{E}_{{\rm{K}}}|}_{l={l}_{1}}=\frac{1}{2}{m}_{i}{v}_{i}^{2}\\ {E}_{{\rm{L}}}=A{c}_{w}\frac{\rho }{2}{v}_{i}^{2}\end{array}\}$$

where *m*_*i*_ is the initial sample mass, *g* is the acceleration due to gravity, *v*_*i*_ is the impact velocity of the sample, *A* is the cross sectional area of sample, *c*_*w*_ is the drag coefficient and *ρ* is sample density. Here, we define *l* = *l*_*i*_ as the distance in flight from the launch position to the position at which impact occurs. Impact velocities were measured by analysis of high-speed video-records. Calculated atmospheric loss energies *E*_L_ lead to a median loss of initial potential energies of about 11.5% for glass bead aggregates and about 15.7% for volcanic ash aggregates, which is in good agreement with atmospheric losses of about 15% reported by others^44^ during drop experiments of larger volcanic clasts.

For each of the 14 aggregate groups displayed on Table 1, 20 drop experiments with 20 aggregates were performed. Impact velocities varied between 1 and 6 m.s^−1^. Maximum fall height *h* (between 0.05 m and 2 m) was chosen according to aggregate stability. If a sample was disintegrated completely, *h* was not increased further. Mass *m*_*i*_ of each sample was evaluated before the fall experiment with a *Sartorius MC 210P* balance with an accuracy of 10^–8^ kg. Aggregates for each experimental setup selected to be in a mass range of ±10 wt%. After impact, the mass *m*_*f*_ of the largest remaining aggregate fragment was evaluated and compared with the initial mass of the aggregate by *m*_*f*_/*m*_*i*_. Based on this ratio, aggregates were categorized into three groups, ‘chipping surface’ (i.e. *m*_*f*_/*m*_*i*_ = 0.9), ‘fragmented’ (i.e. 0.1 < *m*_*f*_/*m*_*i*_ < 0.9) and ‘total disintegration’ (i.e. *m*_*f*_/*m*_*i*_ < 0.1). See Appendix for the complete dataset.

## Results

**Figure 2:**
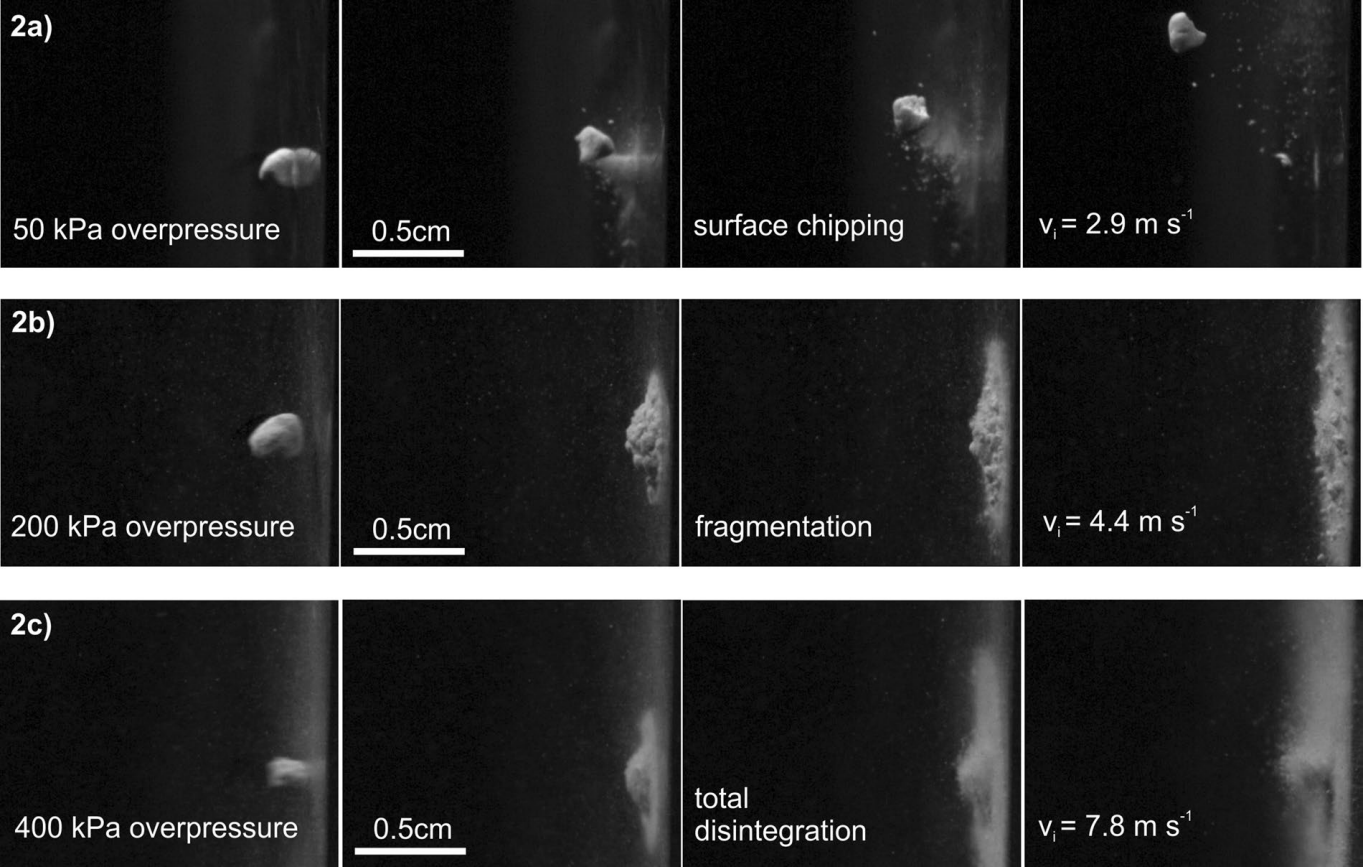
Sequence of photos taken with a high-speed camera; aggregates impact on metal wall of the Aarhus setup. (**a**) Sequence shows surface chipping of aggregate, (**b**) fragmentation and (**c**) total disintegration.

A total of 280 aggregates for the drop experiments were chosen to be consistent in mass to facilitate dataset comparison. On average, the aggregate mass *m*_*i*_ was 1.4 mg with a standard deviation of 0.16 mg. High-speed videos reveal different break-up behavior as a function of impact velocity ranging between 2.9–7.8 m.s^−1^ as summarized in Fig. 2. Experiments with a nozzle overpressure of 50 kPa lead to surface chipping (Fig. 2a), with the aggregate remaining mostly intact after impact. Experiments with 200 kPa overpressure resulted in further fragmentation of the aggregate, with largest fragments showing up to 5 wt.% of the parental aggregate (Fig. 2b). Overpressure experiments of 400 kPa led to total disintegration of the aggregate, no fragments could be observed in the high-speed video or recovered from the ground after the experiment (Fig. 2c).

**Figure 3:**
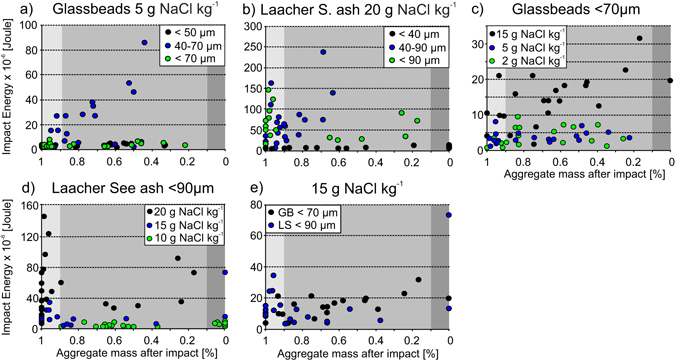
(**a**,**b**) The influence of particle size on aggregate stability. (**c**,**d**) The influence of NaCl binder concentration on aggregate stability. (**d**) The influence of primary particle surface morphology on aggregate stability.

We used our data to evaluate and isolate the effects of binder (NaCl) concentration, particle-size and of particle roughness on the energy required to break-up aggregates. In general, larger primary particle sizes produced stronger aggregates that required higher break up energies. Figure 3a shows glass bead aggregates bound with 5 g.kg^−1^ NaCl. In this dataset, higher impact energies were necessary to fragment aggregates consisting of coarse glass bead sizes (40–70 µm) compared with those consisting of fine glass bead sizes. Equivalent results can be seen for the volcanic ash aggregates (Fig. 3b), in which samples with coarse particle fractions (40–90 µm and <90 µm) needed higher impact energies to fragment than the volcanic ash aggregates of the same salt concentration but a primary particle size distribution of <40 µm. Further, volcanic ash aggregates with coarse primary particles either show surface chipping (left gray field) or fragmentation (center gray field), whereas volcanic ash aggregates with only fine primary particles mainly show total disintegration and at lower impact energies. Overall a clear positive effect of larger particle size distribution on aggregate stability could be observed for both glass bead and volcanic ash aggregates. We report the complete dataset in the Appendix.

In a second experimental suite, we compared the stability of aggregates bound by different NaCl concentrations but having the same primary particle size distribution. Higher NaCl concentrations in the bridges between particles conferred greater stability to the aggregates. Glass bead aggregates of the same primary particle sizes (<70 µm) require higher impact energies in order to breakup if there is more NaCl binding the particles (Fig. 3c). Trends become even more clear for the volcanic ash aggregates (Fig. 3d). Here, not only higher impact energies are required to break aggregates with higher NaCl concentrations, but also the breakup behavior changes. Approximately 75% of the tested samples with high binder concentration (20 g.kg^−1^) exhibit surface chipping upon impact, whereas samples with comparatively low NaCl content (10 g.kg^−1^) undergo fragmentation or total disintegration. We conclude that the salt budget available for particle-particle binding increased aggregate stability against breakup processes.

A final suite of drop experiments was conducted to investigate in the effect of surface morphology of primary particles on aggregate strength. No clear effect was empirically obvious. Stability of glass bead aggregates (spherical primary particles) and volcanic ash aggregates (angular, irregular primary particles) with the same NaCl concentrations do not reveal any difference in the breakup behaviour or impact energies required for breakup (Fig. 3e). We conclude here that particle morphology is a second order control on aggregate stability and that spherical assumptions can be made for the mechanical stability of volcanic ash aggregation.

**Figure 4:**
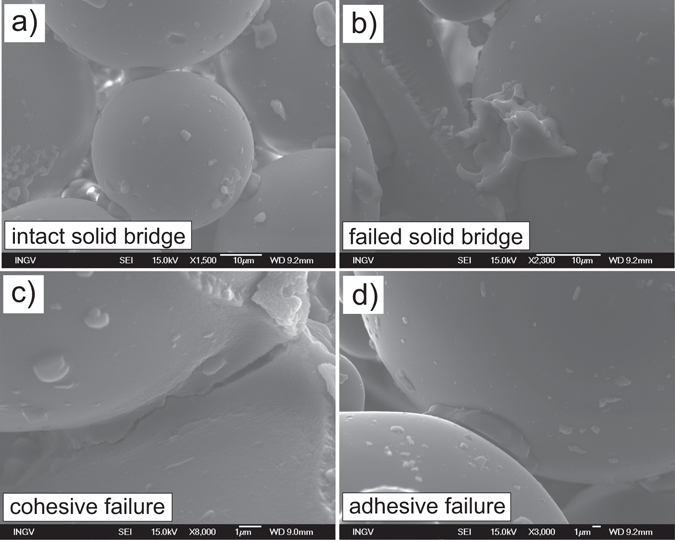
(**a**) An intact solid NaCl bridge connecting two glass beads. (**b**) A failed solid NaCl bridge with the once connected glass bead missing. (**c**) Cohesive failure within a solid NaCl bridge. (**d**) Adhesive failure between a solid NaCl bridge and a glass bead.

**Figure 5:**
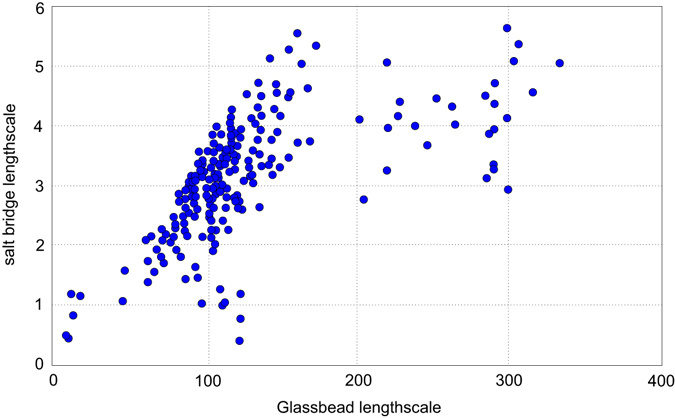
Solid NaCl bridge volumes evaluated from SEM analysis are plotted against total available surface of the two particles connected through the solid bridge. Maximum salt bridge volumes show exponential dependence on available particle surface area.

Scanning electron microscopy (SEM) provides insights into the size and geometry of NaCl crystals as well as their failure mode upon breakup. This analysis shows (i) intact bridges (Fig. 4a), (ii) failed bridges (Fig. 4b), (iii) bridges with cracks (i.e. cohesive failure, Fig. 4c) or (iv) cracks between bridge and solid particle (i.e. adhesive failure, Fig. 4d). Salt bridge volumes were estimated by measuring visible horizontal and vertical axes assuming cuboid growth valid for NaCl crystals. In reality there are deviations from cuboid geometry, conferring minor error on our salt bridge volume estimates. Nevertheless, we observed a positive correlation between salt crystal length and particle radius suggesting that bridge thickness scales with average particle size (Fig. 5).

### Analysis and discussion

Once formed, aggregates can break up in three different micromechanical ways: (1) surface chipping, (2) fragmentation into smaller aggregate pieces, or (3) total disintegration into particle sizes comparable to the primary ones. In volcanic ash plumes, total disintegration, the most efficient process in releasing primary particles back into the plume, could result in re-entrainment of fine ash particles in the plume dispersal trajectory. We show that the mode of aggregate break-up depends on both bulk stability of the aggregate and on the impact energy. Previous discrete element method simulations, as well as experiments, have shown that aggregates impacting a solid wall generate multiple branches of cracks that dynamically propagate inside the aggregate, causing break up of solid bridges between primary particles^45^. The propagation of the cracks depends primarily on the impact energy. While the propagating master cracks cause the aggregate to break-up into fragments, areas of high crack density are also the regions from which primary particles are released.

Impact energies between two aggregates or an aggregate and a clast cannot be easily predicted for volcanic plume conditions. A readily calculable constraint of impact energy for volcanic particles or aggregates in a plume is the energy at terminal velocity v_t_, which depends on the mass of particles or clasts via Eq. 2:

2 $${{\rm{v}}}_{{\rm{t}}}=\sqrt{\frac{2{\rm{mg}}}{{{\rm{c}}}_{{\rm{w}}}{\rm{\rho }}{\rm{A}}}}$$

with the mass m, gravity g, drag coefficient *c*_*w*_, aggregate or particle density ρ and cross sectional area of aggregate or particle A. Terminal fall velocities were reached or exceeded only in our experimental setup 1, and not in setup 2, due to insufficient fall distances. Nevertheless, total disintegration was routinely observed at impact velocities below v_t_.

**Figure 6:**
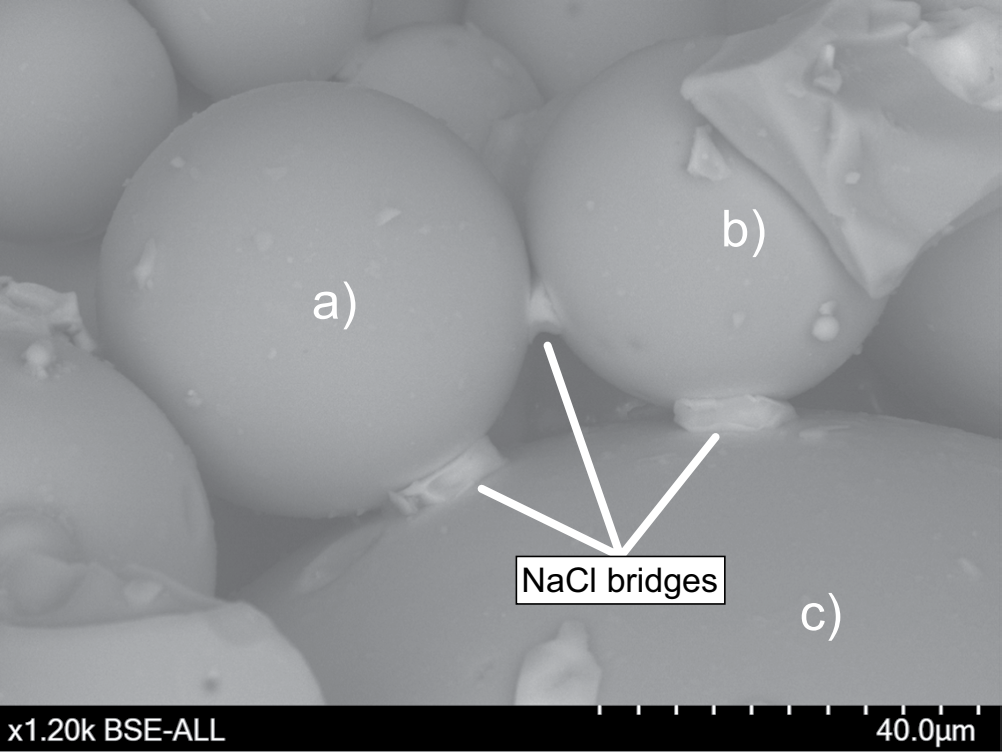
SEM image of aggregated glass beads. The two glass beads (**a**) and (**b**) are connected with each other through a smaller solid NaCl bridge than they are to glass bead (**c**). Glass bead (**c**) is larger in volume and therefore surface and allows for the establishment of more voluminous bridges.

Our experimental results illustrate that aggregates with small primary particle sizes (e.g. <50 µm) are less stable than aggregates with large primary particle sizes (e.g. 40–70 µm). Aggregation in natural volcanic environments rarely exceeds an upper limit in particle size of 200–250 µm^5, 46^; however, primary particles larger than 200 µm have been shown to incorporate in oversaturated (wet) pockets of the solid/liquid mixture, simulating mud drops^10^. Our SEM observations of solid salt bridges cementing analogue glass bead aggregates shows a clear dependence of salt bridge volume on available primary particle surface area (Fig. 5). This relationship can be explained in the context of previous work^41^ that shows how surface liquids such as H_2_O may dissolve salt crystals and create a salt brine. The capillary forces at the contact line between the liquid and the particle cause migration of the salt brine to liquid bonding points between two particles, bunching the liquid in collars around particle-particle contacts. The amount of re-mobilized surficial salt deposits depends on the available catchment area, i.e. the cumulative surface of primary particles. Large, voluminous salt bridges can be formed if the catchment area is significantly large and the number of contact points is small. Two 50 µm glass beads can be cemented by one much larger salt bridge than two glass beads with 10 µm diameter (see also Fig. 6). Generally speaking, the size of salt bridges depends on the connection density, i.e. whether there are many or just few other glass beads connected with each other. This also translates into a higher probability of large salt bridges in aggregates formed from a monodisperse starting particle size distribution and explains why aggregates containing very fine particles (0–40 µm) relative to the mean particle size (highly polydisperse distributions) proved to be comparatively less stable upon impact. In the case in which a large glass bead is connected with many other glass beads, the total available surface salt is probably separated into several contact points, where, at comparable available salt volume, smaller individual salt bridges will crystallize. Salt will also be re-mobilized on ash particles; however, given the more irregular shapes of ash grains, the number of contact points is probably higher than between spheres, producing a relatively weaker connection. However, the effect of complex surface morphologies allows for mechanical interlocking of ash grains adding to the bulk mechanical strength. Indeed, we observed comparable aggregate strengths for ash and glass-bead aggregates with similar binder concentration and particle-size distribution (see Fig. 3).

The strength of aggregates can be calculated based on forces required to separate two particles connected through a solid bridge for a given size, volume and strength. Models, such those proposed by other authors^47, 48^ assume breakage of solid salt bridges to occur at the neck of a bridge – the area with the smallest diameter – which is termed cohesive failure. Contrastingly, adhesive failure occurs by the separation of the contact point between the solid bridge and the primary particle (Fig. 4). The diameter of the bridge neck increases with time as capillary forces drive more brine liquid to the contact zone, bunching up a collar of liquid which, upon drying, precipitates a binding crystal assemblage. Therefore, the force *F* required to separate two particles connected by a solid bridge increases with an increasing neck diameter and can be calculated with a simple micromechanical scaling^21^:

3 $$F\approx \pi {r}_{sb}^{2}{\sigma }_{sb}$$

where *r*_*sb*_ is the radius of the narrowest bridge part (the neck) and *σ*_*sb*_ is the neck strength^21^. Bonding strengths of various salts, *σ*_*sb*_, including NaCl have been experimentally investigated^35^. Average tensile and compressive bonding strengths of crystallized NaCl compounds are reported to be between 0.2 MPa (tensile) and 30 MPa (compressive)^47^. The force required to break the neck region *F*, can then be applied to the model^48^ in which the overall aggregate strength is calculated by scaling neck strengths to the porosity *ε* of the aggregate

4 $${\sigma }_{cr}=z(\varepsilon )\frac{F}{{d}_{p}^{2}}$$

where *d*_*p*_ is the whole aggregate diameter and *z* is a function dependent on porosity *ε*, given in turn by^49^:

5 $$z(\varepsilon )=(1-\varepsilon )/\varepsilon $$

**Figure 7:**
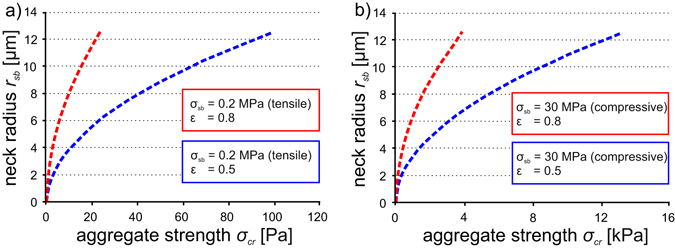
Aggregate strength σ_cr_ of artificial aggregates have been computed, following the models of Johnson *et al*.^49^ and Rumpf^48^. Solid salt bridge neck radii represent SEM analysis results or our artificial aggregates. (**a**) shows aggregate strengths for tensile stress case and aggregate porosities ε of 0.5 and 0.8. (**b**) shows aggregate strengths for compressive stress case and aggregate porosity of 0.5 and 0.8.

Other authors^21^ pointed out that the major challenge in applying micromechanical views of disaggregation processes lies in the uncertainty in neck diameters of the solid bridge. Here, we directly observe maximum bridge length scales and compare them with particle radius (Fig. 5). Using the constraints from our experimental samples, we demonstrate end-member solutions to Eqs 3–5, in which we show how the strength scales with neck radius (100 nm and 12.5 µm evaluated from SEM data, Fig. 5) in some scenarios of two different aggregate porosities of 0.8 and 0.5 which reflect the densities measured for our artificial samples. Lowest aggregate strengths of up to 25 Pa are calculated for the tensile stress and high porosity (0.8) case, whereas the highest aggregate strength of up to 15 kPa can be computed for a compressive stress and the low porosity (0.5) case (Fig. 7). Although the calculated aggregate strengths are based on glass bead aggregates and their solid bridge values, we assume very similar strength values for ash aggregates. Low aggregate strengths (1 mPa to a few Pa) are typical of aggregates with fine primary particle sizes (<40 µm for ash and <50 µm for glass beads), and with high strengths are consistent with aggregates composed of coarse primary particle sizes (up to 90 µm) that are capable of generating larger solid salt bridges. Consequently, one implication is that aggregation of fine ash alone will produce the weakest aggregates and therefore it is fine-grained material is most likely to be disaggregated upon impact and re-suspended in dynamic plumes. Also, accretionary lapilli containing ash particles <40 µm (as used in the experiments) have never been documented in nature which supports the theory that they either don’t survive the plume or they don’t last long enough after sedimentation to be measured.

### Relevance to natural processes and implications

Aggregation models in plume simulations are not based on either the physical description of aggregation growth^17, 18^ or the bulk approach^33, 35, 36, 50^, which is focused on the field deposit. The full description of the aggregation processes confers a high computational cost. To reduce the computational cost, the models are set up to calculate the aggregate contribution and its distribution by pre-aggregating the erupted mixture. While the models are used to fit deposits, they often require tuning via inversion methods to capture the aggregated contribution observed. This implies that the tuning procedure applied in modelling plume aggregation is an approximate method of accounting for the bulk effect of aggregation and disaggregation. A full implementation of disaggregation within tephra dispersal models requires a constitutive understanding of how aggregation and post-aggregation disaggregation occurs during eruptions. This study aims to highlight the feasibility of developing a first such constitutive law for disaggregation impact energies at experimental Reynolds numbers <500 (ref. 12), velocity of the bulk airflow 0.15–0.22 m/s (ref. 12), environmental temperature of 40–60 °C, and bulk ash density 900 kg/m^3^. At these conditions, impacts lead to surface chipping, fragmentation and total disintegration within the plume. To go towards the full characterization of the tephra transport and its sedimentation, it would be useful to assess disaggregation rates in real meteorological conditions (e.g. wind, temperature, air moisture profiles) and at real mass eruption rates, eruption durations and airborne ash mass.

While this study highlights the importance of considering aggregation processes within a model of plume dispersion and sedimentation, we have demonstrated that aggregates can disintegrate if impact energies are sufficient^3^. Other studies^15^ indeed show that tephra fall deposits from the 2010 Eyjafjallajökull eruption (Iceland) were found to be enriched with fine ash in proximal areas, in combination with deposited aggregates, suggesting that upon aggregation-induced sedimentation from the plume, aggregate break-up on impact skewed the particle size distributions measured.

In this study, we have presented insights into the influence of primary particle size distribution, surface morphology and binder concentration on aggregate stability. Other authors^12^ have additionally described the effect of primary particle size distribution, effective particle concentration, humidity and binder concentration on aggregation efficiency. Together these experimental datasets illustrate that fine particles (e.g. ash <40 µm) are much more efficient (up to 100%) in their aggregation rate than coarse particles (e.g. ash 40–90 µm), but are also much more prone to subsequent break-up due to their low comparative stability.

## Conclusion

This study evaluated the stability of aggregates produced artificially from analogue soda-lime silicate glass beads and natural volcanic ash. NaCl was used as binding agent. Impact experiments demonstrated the influence of (1) primary particle size distribution, (2) particle surface area and morphology and (3) binder concentration on aggregate stability. Salt bridge volumes of glass bead aggregates obtained via Scanning Electron Microscopy were used for numerical calculations of aggregate strength, computed to be in the range of <1 Pa up to several 100 Pa. Notably, coarse-grained aggregates (made of primary particles >50 µm) exhibit a significantly increased stability compared with fine-grained aggregates. Aggregates with small primary particle size (<50 µm) are up to one order of magnitude weaker than aggregates with larger primary particle size (>50 µm). Current tephra dispersal models regard aggregation of ash as irrevocable leading to sedimentation and removal from the atmosphere^19^ without explicitly estimating the disaggregation contribution. Here, we have presented quantitative data on disaggregation that can be used for further work investigating potential links with eruption source parameters which are required for numerical ash plume dispersal forecasting. In combination with other experimental studies, it is now possible to attribute disaggregation processes to eruption parameters such as mass eruption rate, eruption intensity, total grain-size distribution, degassing rate and magma composition, or to meteorological parameters such as air moisture and temperature, or to other particle parameters such as primary particle size distribution, effective particle concentration, binder concentration, Reynolds number of the solid/gas system and exposure time.

## Electronic supplementary material

Supplementary Dataset 1
